# Development and Internal Validation of Novel Nomograms Based on Benign Prostatic Obstruction-Related Parameters to Predict the Risk of Prostate Cancer at First Prostate Biopsy

**DOI:** 10.3389/fonc.2018.00438

**Published:** 2018-10-16

**Authors:** Luigi Cormio, Luca Cindolo, Francesco Troiano, Michele Marchioni, Giuseppe Di Fino, Vito Mancini, Ugo Falagario, Oscar Selvaggio, Francesca Sanguedolce, Francesca Fortunato, Luigi Schips, Giuseppe Carrieri

**Affiliations:** ^1^Department of Urology and Renal Transplantation, University of Foggia, Foggia, Italy; ^2^Department of Urology, ASL, Chieti, Italy; ^3^Department of Urology, SS Annunziata Hospital, “G.D'Annunzio” University of Chieti, Chieti, Italy; ^4^Department of Pathology, University of Foggia, Foggia, Italy; ^5^Department of Medical and Surgical Sciences, University of Foggia, Foggia, Italy

**Keywords:** prostate biopsy, prostate cancer, nomogram, lower urinary tract symptoms, prostate volume

## Abstract

The present study aimed to determine the ability of novel nomograms based onto readily-available clinical parameters, like those related to benign prostatic obstruction (BPO), in predicting the outcome of first prostate biopsy (PBx). To do so, we analyzed our Internal Review Board-approved prospectively-maintained PBx database. Patients with PSA>20 ng/ml were excluded because of their high risk of harboring prostate cancer (PCa). A total of 2577 were found to be eligible for study analyses. The ability of age, PSA, digital rectal examination (DRE), prostate volume (PVol), post-void residual urinary volume (PVR), and peak flow rate (PFR) in predicting PCa and clinically-significant PCa (CSPCa)was tested by univariable and multivariable logistic regression analysis. The predictive accuracy of the multivariate models was assessed using receiver operator characteristic curves analysis, calibration plot, and decision-curve analyses (DCA). Nomograms predicting PCa and CSPCa were built using the coefficients of the logit function. Multivariable logistic regression analysis showed that all variables but PFR significantly predicted PCA and CSPCa. The addition of the BPO-related variables PVol and PVR to a model based on age, PSA and DRE findings increased the model predictive accuracy from 0.664 to 0.768 for PCa and from 0.7365 to 0.8002 for CSPCa. Calibration plot demonstrated excellent models' concordance. DCA demonstrated that the model predicting PCa is of value between ~15 and ~80% threshold probabilities, whereas the one predicting CSPCa is of value between ~10 and ~60% threshold probabilities. In conclusion, our novel nomograms including PVR and PVol significantly increased the accuracy of the model based on age, PSA and DRE in predicting PCa and CSPCa at first PBx. Being based onto parameters commonly assessed in the initial evaluation of men “prostate health,” these novel nomograms could represent a valuable and easy-to-use tool for physicians to help patients to understand their risk of harboring PCa and CSPCa.

## Introduction

Prostate biopsy (PBx) is the standard method for diagnosing prostate cancer (PCa) but the diagnostic yield of this procedure remains low. In current clinical practice the cancer detection rate (CDR) of a first extended PBx prompted by an elevated serum prostate-specific antigen (PSA) level and/or an abnormal digital rectal examination (DRE) is around 40% (1), dropping to approximately 25% in the setting of screening programs, i.e., patients with serum PSA between 2.5 and 10 ng/mL (2).

In the last 20 years, efforts to improve the diagnostic yield of PBx have been oriented toward the construction of predictive models combining serum PSA and DRE findings with other clinical information such as age, prostate volume (PVol), %free PSA, etc., as well as toward the development of novel biomarkers or imaging techniques. A recent meta-analysis (3) demonstrated that some but not all the most common PCa risk prediction models perform better than serum PSA in predicting PCa diagnosis. Novel biomarkers, such as the precursor isoform [-2]proPSA (p2PSA) and the Prostate Cancer Antigen 3 (PCA3), also perform better than serum PSA and have further, but not dramatically, increased the accuracy of PCa risk prediction models (4, 5). Multiparametric magnetic resonance imaging (mpMRI) of the prostate has also been suggested to improve PBx diagnostic yield; however, it does not dramatically increase the accuracy of PCa risk prediction models (6–8) and is not recommended in the setting of first PBx (9).

In this scenario, the identification of cheap, non-invasive and readily available clinical data that could improve the accuracy of PCa risk prediction models represents a major clinical issue. Recently, we demonstrated that an elevated post-void residual urinary volume (PVR) and the absence of bladder outlet obstruction (BOO), as assessed by a peak flow rate (PFR) of >10 mL/s, are independent predictors of PBx outcome (10, 11). Since these simple non-invasive parameters are commonly assessed in the initial evaluation of men “prostate health,” in the present study we aimed to assess whether the addition of PFR and PVR to a multivariate logistic regression model based on standard clinical parameters (age, serum PSA, DRE, and PVol) could increase the model predictive accuracy.

## Patients and methods

The study protocol was approved by the University of Foggia Ethics Committee and was carried out in agreement with the provisions of the Declaration of Helsinki. Written informed consent to take part was given by all participants. Data of patients scheduled for ultrasound-guided transrectal PBx because of increased serum PSA (≥4 ng/mL) and/or abnormal DRE were prospectively entered into our dedicated Institutional Review Board-approved database. All patients underwent PSA measurement before DRE and transrectal ultrasound (TRUS). Uroflowmetry (UFM) was carried out before PBx, waiting for the patient to report a strong sensation to void. Following local non-infiltrative anesthesia (12, 13), TRUS was used to determine prostate and transition zone volume and to guide transrectal prostate sampling according to our systematic 18-core biopsy scheme (14).

Men receiving 5 alfa-reductase inhibitors (5-ARIs), or who had previously undergone PBx or invasive treatment for benign prostatic hyperplasia, or with dwelling urethral catheters, or with a voided volume of less than 150 ml were excluded from the present study. Patients with PSA>20 ng/ml were also excluded as we found them to have a too high risk (>75%) of harboring PCa.

A senior uropathologist blind to PFR and PVR data evaluated the specimens according to contemporary diagnostic criteria for high-grade prostatic intraepithelial neoplasia (HGPIN), atypical small acinar proliferation (ASAP) of prostate, and PCa. Patients diagnosed with HGPIN or ASAP were excluded from the present analysis.

Outcomes of interest were the rate of all PCas and the rate of clinically significant prostate cancers (CSPCa) defined as those with a Gleason Grade Group (GGG) >1 according to the International Society of Urological Pathology (ISUP) consensus (15, 16).

### Statistical analysis

Continuous variables are reported as medians; they were compared by the Mann Whitney test for independent groups. Differences in rates were tested by the chi square test.

The value of the different clinical variables in predicting PCa and CSPCa was assessed by uni- and multi- variable binary logistic regression analyses. Receiver operator characteristic (ROC) curves analysis was used to test the predictive accuracy of multivariate logistic regression models including the various clinical variables and the areas under the ROC curves were compared by a non-parametric approach (17).

Two nomograms were then built based on the coefficients of the logit function. Observed vs. predicted values were plotted to evaluate for calibration by using the locally weighted scatter plot smoothing method. Finally, internal validation was performed using the leave-one-out cross-validation (LOOCV). The linear prediction of the logistic function, adjusted after internal validation, was used to compute the AUC of the model, to graphically assess calibration and to perform the decision curve analysis (DCA).

Statistical analyses were performed using Stata 12 (StataCorp LP, College Station, TX, USA). All tests were 2-sided with a significance level set at *p* < 0.05.

## Results

### Baseline characteristics

Between January 2006 and May 2017, a total of 3,461 patients underwent TRUS-guided PBx at our Institution; 2,577 met the inclusion criteria. Of all, 1,018 (39.5%) patients were diagnosed with PCa of any ISUP Grade Group. Within PCa patients, 612 were considered clinically significant (ISUP Grade Group >1).

Descriptive analyses showed that patients diagnosed with cancer were older (68 vs. 65 years old) and with higher rates of suspicious DRE (53.7 vs. 33.8%) than their counterpart without cancer. Moreover, higher PSA level (7 vs. 6 ng/ml), smaller prostate volume (42 vs. 60 ml), lower PVR (20 vs. 40 ml) and higher PFR (13 vs. 12 ml/s) were showed in patients with cancer as compared to those without cancer. Results were virtually the same when ISUP 1 and ISUP >1 patients were compared to those without PCa (Table 1).

**Table 1:** Patients descriptive characteristics.

**Variables**	**No Cancer (*N* = 1,559)**	**PCa (*N* = 1,018)**	***p*-value**	**ISUP 1 PCa (*N* = 406)**	*p*-value^*^	**ISUP>1 PCa (*N* = 612)**	*p*-value ^**^
Age (years)	65 (60, 70)	68 (63, 74)	<**0.0001**	67 (62, 72)	**0.001**	70 (65, 75)	<**0.0001**
Suspicious DRE	33.8% (527)	53.7% (547)	<**0.0001**	39.8%(162)	0.065	62.8% (384)	<**0.0001**
PSA (ng/mL)	6 (5, 8)	7 (5, 10)	**0.0002**	6 (5, 8)	**0.041**	7 (5, 11)	<**0.0001**
PVol (mL)	60 (44, 80)	42 (32, 57)	<**0.0001**	47 (35, 61)	<**0.0001**	40 (30, 55)	<**0.0001**
PVR (mL)	40 (20, 70)	20 (1, 50)	<**0.0001**	20 (1, 50)	<**0.0001**	22 (1, 50)	<**0.0001**
PFR (mL/s)	12 (8, 16)	13 (10, 17)	<**0.0001**	14 (10, 18)	<**0.0001**	13 (9, 17)	<**0.0001**

*Continuous variables are reported as medians (interquartile range); categorical variables are reported as rates (n)*.

* *ISUP 1 vs. no cancer*.

** *ISUP >1 vs. no cancer. DRE, digital rectal examination; ISUP, International Society of Urological Pathology; PCa, prostate cancer; PFR, peak flow rate; PSA, prostate-specific antigen; PVol, prostate volume; PVR, post-void residual urinary volume. The bold values are the statistically significant differences*.

Univariable binary logistic regression analysis demonstrated that all clinical variables predicted PCa (Table 2) and CSPCa (Table 3), but PFR failed to confirm its predictive value in multivariable binary logistic regression analysis (Tables 2, 3).

**Table 2:** Univariable and multivariable binary logistic regression analysis testing the value of clinical variables in predicting Prostate Cancer (any ISUP).

**Variables**	**Univariable analysis**			**Multivariable analysis**		
	**Odds ratio (95% C.I.)**	**Std. Err**.	***P* > |*z*|**	**Odds ratio (95% C.I.)**	**Std. Err**.	***P* > |*z*|**
Age (years)	1.057 (1.046–1.069)	0.006	<**0.001**	1.076 (1.059–1.093)	0.009	<**0.001**
Suspicious DRE	2.277 (1.857–2.791)	0.237	<**0.001**	1.852 (1.472–2.329)	0.217	<**0.001**
PSA (ng/mL)	1.061 (1.038–1.084)	0.012	<**0.001**	1.093 (1.055–1.132)	0.020	<**0.001**
PVol (mL)	0.970 (0.966–0.974)	0.002	<**0.001**	0.971 (0.966–0.976)	0.003	<**0.001**
PVR (mL)	0.989 (0.987–0.991)	0.001	<**0.001**	0.993 (0.989–0.996)	0.002	<**0.001**
PFR (mL/s)	1.031 (1.019–1.044)	0.006	<**0.001**	1.010 (0.992–1.028)	0.009	0.271

*DRE, digital rectal examination; ISUP, International Society of Urological Pathology; PCa, prostate cancer; PFR, peak flow rate; PSA, prostate-specific antigen; PVol, prostate volume; PVR, post-void residual urinary volume. The bold values are the statistically significant differences*.

**Table 3:** Univariable and multivariable binary logistic regression analysis testing the value of clinical variables in predicting clinically significant Prostate Cancer (ISUP>1).

**Variables**	**Univariable analysis**			**Multivariable analysis**		
	**Odds ratio (95% C.I.)**	**Std. Err**.	***P* > |*z*|**	**Odds ratio (95% C.I.)**	**Std. Err**.	***P* > |*z*|**
Age (years)	1.077 (1.063–1.091)	0.007	<**0.001**	1.092 (1.072–1.113)	0.011	<**0.001**
Suspicious DRE	3.143 (2.467–4.005)	0.388	<**0.001**	2.701 (2.069–3.527)	0.368	<**0.001**
PSA (ng/mL)	1.120 (1.093–1.147)	0.014	<**0.001**	1.155 (1.112–1.201)	0.023	<**0.001**
PVol (mL)	0.969 (0.964–0.974)	0.002	<**0.001**	0.970 (0.964–0.977)	0.003	<**0.001**
PVR (mL)	0.992 (0.990–0.995)	0.001	<**0.001**	0.994 (0.990–0.998)	0.002	**0.006**
PFR (mL/s)	1.021 (1.007–1.035)	0.007	**0.002**	1.003 (0.983–1.024)	0.010	0.752

*DRE, digital rectal examination; ISUP, International Society of Urological Pathology; PCa, prostate cancer; PFR, peak flow rate; PSA, prostate-specific antigen; PVol, prostate volume; PVR, post-void residual urinary volume. The bold values are the statistically significant differences*.

**Figure 1:**
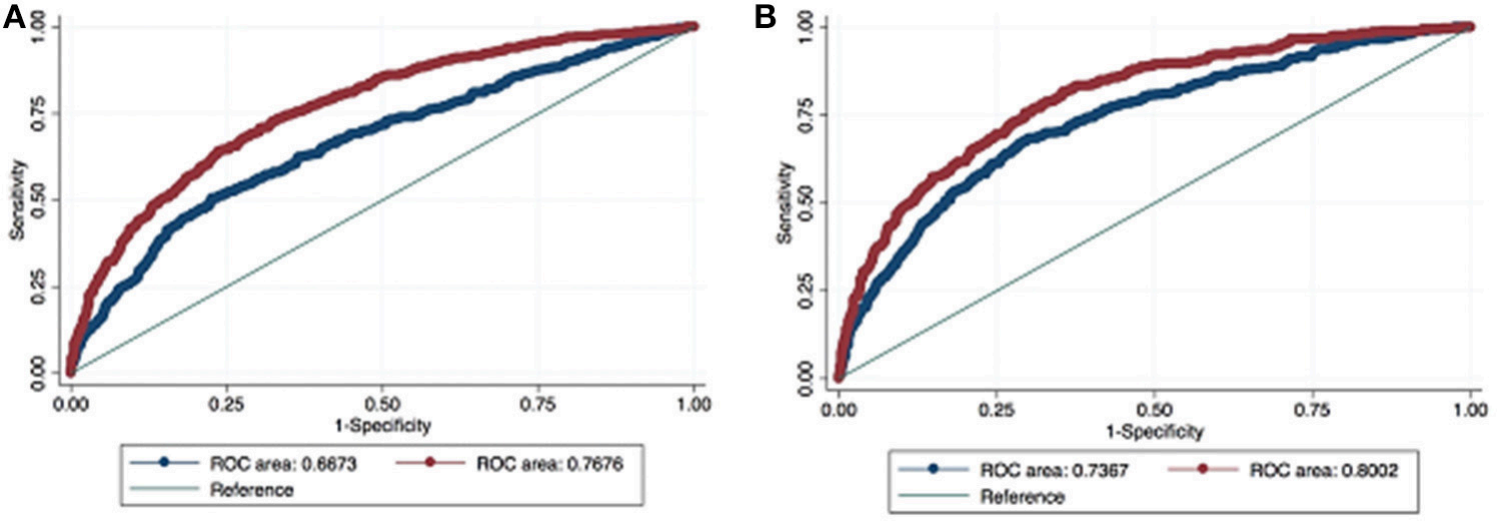
Receiver operating characteristic (ROC) curve analysis comparing base model (red line = age, PSA, and DRE) with the full model (blue line = age, PSA, DRE, PVol, PVR) in predicting prostate cancer **(A)** and clinically significant prostate cancer **(B)**.

**Figure 2:**
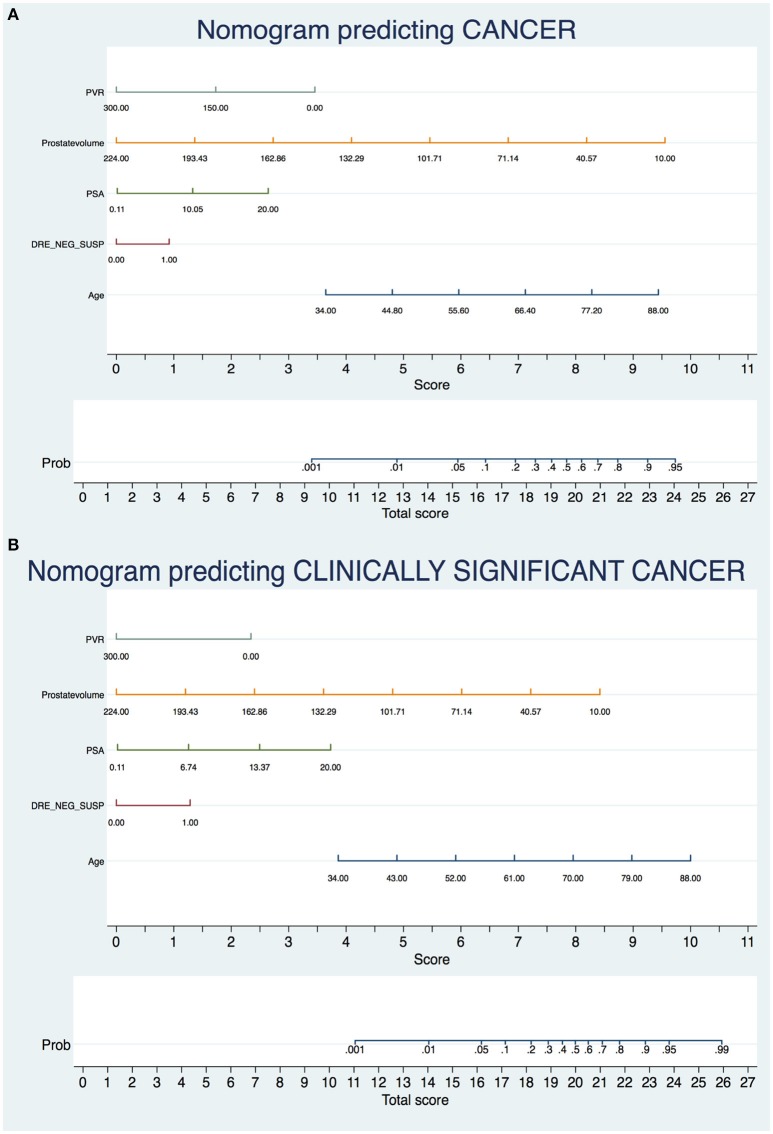
Nomograms predicting prostate cancer **(A)** and clinically significant prostate cancer **(B)**.

ROC curve analysis demonstrated that the addition of the BPO-related parameters PVol and PVR increased the AUC of the model based on standard variables (age, PSA and DRE status) from 0.664 to 0.768 in predicting PCa and from 0.7365 to 0.8002 in predicting CSPCa (Figure 1). The beta coefficients of the logit function of these models (Supplementary Tables 1, 2) were then used to construct the nomograms to predict PCa and CSPCa (Figures 2A,B).

**Figure 3:**
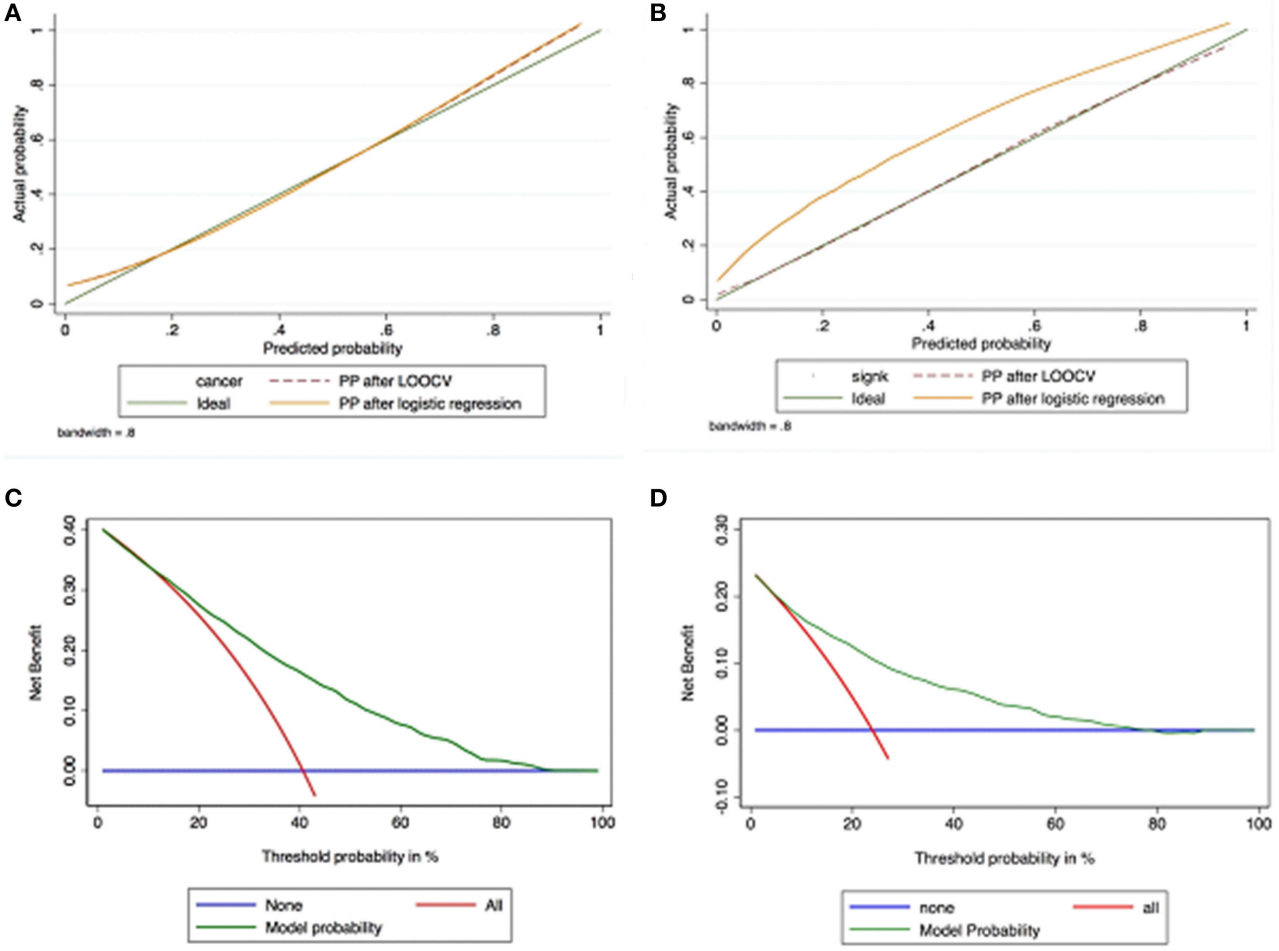
Calibration plot of observed vs. predicted probabilityof PCa **(A)** and CSPCa **(B)** after leave-one-out cross validation, demonstrating excellent concordance. Decision curve analyses demonstrating net benefit between the threshold probabilities of ~15 and ~80% for the model predicting PCa **(C)** and between the threshold probabilities of ~10 and ~60% for the model predicting CSPCA **(D)**.

Calibration plot of observed vs. predicted probability of PCa (Figure 3A) and CSPCa (Figure 3B) after leave-one-out cross validation, demonstrated excellent concordance. DCA demonstrated the net benefit associated with the use of the model-derived probability for predicting PCa (Figure 3C) and CSPCa (Figure 3D); the model predicting PCa was of value between ~15 and ~80% threshold probabilities, whereas the one predicting CSPCa was of value between ~10 and ~60% threshold probabilities. Finally, an online risk calculator was developed based on the two nomograms (http://www.foggiaprostatecancerriskcalculator.com/calc/).

## Discussion

Current European Association of Urology guidelines warn about the necessity to offer an individualized risk-adapted strategy for early PCa detection and highlight the importance of patient participation into the decision-making process when indication to biopsy is placed (9). The present study sought to develop a novel instrument based onto readily-available clinical parameters that can help the physician to explain the risk of each single patient to harbor PCa and CSPCa. We found that the addition of the BPO-related variables PVol and PVR to a model based on age, PSA and DRE findings increased the model predictive accuracy from 0.664 to 0.768 for PCa and from 0.7365 to 0.8002 for CSPCa. Turning findings into clinical practice, in a 70 years old man with PSA 6 ng/ml and normal DRE, the estimated probability of harboring PCa is 27% in case of PVol 60 ml and PVR 80 ml, as opposed to 63% in case of PVol 30 ml and PVR 0 ml; the estimated probability of harboring CSPCa in the above-mentioned conditions is 11 and 33%, respectively.

For more than 20 years (18), it has been shown that the ability of the PSA test to discriminate malignant from benign prostate is dramatically lower in men with lower urinary tract symptoms (LUTS). Indeed, several studies pointed out that PVol, which is directly correlated to BPO and LUTS, is inversely correlated with the risk of harboring PCa in men scheduled for PBx (19, 20).

As mentioned above, we demonstrated that two simple, noninvasive, and objective clinical parameters potentially related to symptomatic BPO such as PFR and PVR were able to independently predict the risk of being diagnosed with PCa in patients scheduled for PBx because of increased PSA levels and/or abnormal DRE (10, 11), thus providing grounds for evaluating them in the setting of a “easy to use” predictive tool such as a nomogram. The present study failed to confirm the role of PFR as an independent risk factor for PCa but confirmed, that PVol and PVR had a high predictive value. The AUC and the calibration plot of our novel nomograms validated them as high performance tools. DCA showed the models being effective against a wide range of threshold probabilities; in other words, the nomograms allow predicting probabilities that apply to most candidates to first PBx with good performances.

The present study also provides support to the inverse relationship between two proxy of intra-prostatic reflux/inflammation, such as large PVol and large PVR, and the risk of being diagnosed with PCa. These findings are in agreement with those of Moreira et al. (21) who evaluated a cohort of 6,132 having undergone repeat PBx after negative baseline PBx in the Reduction by Dutasteride of prostate Cancer Events (REDUCE) study. They showed that chronic prostate inflammation alone or in combination with prostate atrophy was associated with both lower risk of PCa and lower risk of high-grade PCa in 2-year repeat biopsy (21). It should however be acknowledged that others showed a correlation between inflammation and development of PCa as the result of the effect of the synergic action of cytokines, oxygen reactive species, and DNA damage (22).

Independently on speculation regarding the role of inflammation in BPH and PCa, the proposed nomograms are based onto simple clinical parameters recommended as first-level exams in the evaluation of patients with LUTS, thus avoiding the use of other more complex and not easily available exams. In a more historical analysis based on an Italian institutional cohort, Guazzoni et al. showed that in patient with a total PSA between 2 and 10 ng/ml, Prostate Health Index (PHI) AUC was 0.76 (23). Also, the PCA3 test has been suggested to increase the predictive accuracy of a “base” model including age, PSA, DRE, PVol, and previous PBx. In patients with PSA < 50 ng/mL, the gain in the AUC ranged from 2 points for continuously coded PCA3 scores to 4 points when a cut-off of 17 score was used (24). Again, this is not a greater gain than the one provided by the addition of PVR, a much cheaper, simpler and commonly available clinical parameter. However, a commercially available assay combining serum PSA with urinary PCA3 and the urinary transmembrane protease, serine 2:v-ets erythroblastosis virus E26 oncogene homolog (TMPRSS2:ERG fusion) provides a 90% specificity and 80% sensitivity in diagnosing PCa (25).

Research is moving toward the identification of novel, potentially simple clinical parameters that could increase our ability to detect PCa while reducing the number of “un-necessary” PBxs. We recently demonstrated that, in a small cohort of 40 patients scheduled for repeat PBx, Pentraxin 3 significantly outperformed PSA (AUC 0.92 vs. 0.55) in predicting the risk of being diagnosed with PCa (26). These findings await validation in a large series of patients scheduled for first PBx.

Strong points of our study include its prospective nature, being one of largest cohorts available in this kind of study, and the use of a standardized extended PBx scheme as well as of a standardized protocol for UFM and PVR. Potential limitations include being a single-center study including only Caucasian white men, with no Africans and Hispanics, and not having recorded data regarding family history associated with PCa diagnosis.

## Conclusion

In men scheduled for first PBx, the risk of harboring PCa and CSPCa is inversely related to the BPO-related parameters PVol and PVR. The addition of these parameters to the base model including age, PSA and DRE significantly increases the models' accuracy in predicting PCa and CSPCa. The resulting nomogram/risk calculator based onto parameters commonly assessed in the initial evaluation of men “prostate health” could represent a valuable and easy-to-use tool for physicians to help patients to understand their risk of harboring PCa and CSPCa.

## Author contributions

LCo and LCi designed the study, carried out the analysis, and wrote the manuscript. FT collected the data and wrote the manuscript. FF and FS analyzed the results and wrote the manuscript. GC and LS supervised the study, analyzed the results, and edited the manuscript. MM, GD, UF, VM and OS wrote the manuscript.

### Conflict of interest statement

The authors declare that the research was conducted in the absence of any commercial or financial relationships that could be construed as a potential conflict of interest.
