# Supplementary material for: Development and Internal Validation of Novel Nomograms Based on Benign Prostatic Obstruction-Related Parameters to Predict the Risk of Prostate Cancer at First Prostate Biopsy
Source: Front Oncol. 2018 Oct 16;8:438. doi: 10.3389/fonc.2018.00438 (PMC6198078; doi:10.3389/fonc.2018.00438)
Supplement: Supplementary file 1 [file Table_1.DOCX]

**Supplementary table 1. Beta coefficients of the logit function to compute the linear prediction.**

**MODEL C predictingdiagnosis prostate cancer.**

| Covariate | Coef. | 95% Conf. Interval | P>\|z\| |
| --- | --- | --- | --- |
| Age |  |  |  |
| per unit | 0.072 | (0.056 to 0.087) | <0.001 |
| DRE_NEG_Y/N |  |  |  |
| per unit | 0.613 | (0.384 to 0.842) | <0.001 |
| PSA |  |  |  |
| per unit | 0.088 | (0.053 to 0.124) | <0.001 |
| Prostate volume |  |  |  |
| per unit | -0.030 | (-0.035 to -0.024) | <0.001 |
| RPM |  |  |  |
| per unit | -0.008 | (-0.011 to -0.004) | <0.001 |
| Constant | -4.121 | (-5.177 to -3.064) | <0.001 |

$$logit\left( \hat{\pi}_{i} \right)=\ln\left( \frac{\hat{\pi}_{i}}{1-\hat{\pi}_{i}} \right)=0.072\left( AGE \right)+0.613\left( DRE \right)+0.088\left( PSA \right)-0.030\left( PROSTATE VOLUME \right)-0.008 \left( RPM \right)-4.121$$

**Supplementary table 2. Beta coefficients of the logit function to compute the linear prediction.**

**MODEL C predicting diagnosis of clinically significant prostate cancer.**

| Covariate | Coef. | 95% Conf. Interval | P>\|z\| |
| --- | --- | --- | --- |
| Age |  |  |  |
| per unit | 0.088 | (0.069 to 0.107) | <0.001 |
| DRE_NEG_Y/N |  |  |  |
| per unit | 0.992 | (0.726 to 1.259) | <0.001 |
| PSA |  |  |  |
| per unit | 0.144 | (0.106 to 0.183) | <0.001 |
| Prostate volume |  |  |  |
| per unit | -0.030 | (-0.037 to -0.024) | <0.001 |
| RPM |  |  |  |
| per unit | -0.006 | (-0.010 to -0.002) | 0.004 |
| Constant | -6.822 | (-8.131 to -5.513) | <0.001 |

$$logit\left( \hat{\pi}_{i} \right)=\ln\left( \frac{\hat{\pi}_{i}}{1-\hat{\pi}_{i}} \right)=0.088\left( AGE \right)+0.992\left( DRE \right)+0.144\left( PSA \right)-0.030\left( PROSTATE VOLUME \right)-0.006 \left( RPM \right)-6.822$$
